# High-Throughput Transcriptomics of *Celf1* Conditional Knockout Lens Identifies Downstream Networks Linked to Cataract Pathology

**DOI:** 10.3390/cells12071070

**Published:** 2023-04-01

**Authors:** Archana D. Siddam, Matthieu Duot, Sarah Y. Coomson, Deepti Anand, Sandeep Aryal, Bailey A. T. Weatherbee, Yann Audic, Luc Paillard, Salil A. Lachke

**Affiliations:** 1 Department of Biological Sciences, University of Delaware, Newark, DE 19716, USA; 2 CNRS, IGDR (Institut de Génétique et Développement de Rennes), Univ. Rennes, UMR 6290, Rennes, F-35000 Rennes, France; 3 Center for Bioinformatics and Computational Biology, University of Delaware, Newark, DE 19716, USA

**Keywords:** lens, eye, cataracts, RNA-binding protein, Cugbp1, development, post-transcriptional control, transcriptome, RNA-sequencing, microarrays

## Abstract

Defects in the development of the ocular lens can cause congenital cataracts. To understand the various etiologies of congenital cataracts, it is important to characterize the genes linked to this developmental defect and to define their downstream pathways that are relevant to lens biology and pathology. Deficiency or alteration of several RNA-binding proteins, including the conserved RBP Celf1 (CUGBP Elav-like family member 1), has been described to cause lens defects and early onset cataracts in animal models and/or humans. Celf1 is involved in various aspects of post-transcriptional gene expression control, including regulation of mRNA stability/decay, alternative splicing and translation. *Celf1* germline knockout mice and lens conditional knockout (*Celf1*^cKO^) mice develop fully penetrant cataracts in early postnatal stages. To define the genome-level changes in RNA transcripts that result from *Celf1* deficiency, we performed high-throughput RNA-sequencing of *Celf1*^cKO^ mouse lenses at postnatal day (P) 0. *Celf1*^cKO^ lenses exhibit 987 differentially expressed genes (DEGs) at cut-offs of >1.0 log2 counts per million (CPM), ≥±0.58 log2 fold-change and <0.05 false discovery rate (FDR). Of these, 327 RNAs were reduced while 660 were elevated in *Celf1*^cKO^ lenses. The DEGs were subjected to various downstream analyses including iSyTE lens enriched-expression, presence in Cat-map, and gene ontology (GO) and representation of regulatory pathways. Further, a comparative analysis was done with previously generated microarray datasets on *Celf1*^cKO^ lenses P0 and P6. Together, these analyses validated and prioritized several key genes mis-expressed in *Celf1*^cKO^ lenses that are relevant to lens biology, including known cataract-linked genes (e.g., *Cryab*, *Cryba2*, *Cryba4*, *Crybb1*, *Crybb2*, *Cryga*, *Crygb*, *Crygc*, *Crygd*, *Cryge*, *Crygf*, *Dnase2b*, *Bfsp1*, *Gja3*, *Pxdn*, *Sparc*, *Tdrd7*, etc.) as well as novel candidates (e.g., *Ell2* and *Prdm16*). Together, these data have defined the alterations in lens transcriptome caused by Celf1 deficiency, in turn uncovering downstream genes and pathways (e.g., structural constituents of eye lenses, lens fiber cell differentiation, etc.) associated with lens development and early-onset cataracts.

## 1. Introduction

Morphogenesis of the vertebrate ocular lens has been studied for over 100 years [1]. In addition to uncovering key principles in developmental biology, understanding the process of lens formation has helped identify genetic causes underlying human lens defects, such as congenital cataracts [2,3]. Indeed, thus far, several regulatory pathways involved in lens development have been identified [4]. While the majority of these studies were focused on signaling and transcriptional regulation [4,5], research over the past ~10 years has shown that RNA-binding protein (RBP)-based post-transcriptional control of gene expression plays key roles in lens development [6,7]. These findings have shown that the expressions of several RBPs, namely, Caprin2, Celf1 (Cugbp1), Rbm24 and Tdrd7, are conserved in lens development across multiple vertebrate species [8,9,10,11,12,13,14,15,16]. Deficiency or mutation in these RBPs in animal models or humans are associated with eye and/or lens defects/cataracts [17,18,19,20,21,22,23]. Functional studies have indicated that these RBPs have a distinct role in spatiotemporal control over key factors in lens development. However, compared to our understanding of signaling and transcription, our knowledge on lens regulatory networks impacted by perturbation of these RBPs is limited.

Celf1 has three RNA-recognition motifs (RRMs) that allow it to bind to its target RNAs and is known to mediate RNA localization, decay/stability, alternative splicing and translation [24,25,26,27]. It has been shown that in the majority of the cases, binding of Celf1 protein to its target mRNA results in the destabilization of the latter [28]. Previously, we demonstrated that *Celf1* germline knockout (KO) or conditional KO (cKO) in the lens results in fully penetrant congenital cataracts in mice. Celf1-knockdown in fish and frogs also results in lens defects, suggesting that Celf1 plays an important role in vertebrate lens development [9]. We previously characterized specific aspects of Celf1 deficiency-based lens defects in mice, demonstrating that Celf1-mediated negative control at the translational level over the cyclin-D kinase inhibitor p27^Kip1^ was important for achieving optimal phosphorylation of nuclear lamin proteins, which in turn is critical for fiber cell nuclear envelope breakdown in normal lens development. This, in addition to Celf1′s positive control of mRNA expression levels of the nuclease Dnase2b, was found to be necessary for nuclear degradation in fiber cells [9]. Subsequently, we showed that Celf1 also played a role in achieving proper protein levels and spatiotemporal distribution of key transcription factors (TFs) in the lens. Indeed, Celf1 was found to be necessary for restriction of the expression of Prox1 protein to fiber cells and that of Pax6 to the anterior epithelium of the lens (AEL), as well as early fiber differentiating cells in normal lens development [10].

While these studies have uncovered specific aspects of Celf1 function in the lens, high-throughput RNA-sequencing (RNA-seq)-based transcriptome analyses of Celf1-deficient mouse lenses has not been described. Such an analysis will identify, on the genome-level, different mRNAs that are altered upon Celf1 deficiency, shedding further light on Celf1′s role in lens development and offering new explanations regarding how alterations in its downstream pathways may contribute to lens pathology in *Celf1*^cKO^ mice. In the present study, we address this critical knowledge gap by performing RNA-seq analysis on newborn lenses from *Celf1*^cKO^ mice and identifying cohorts of differentially expressed genes (e.g., *Cryab*, *Cryba2*, *Cryba4*, *Crybb1*, *Crybb2*, *Cryga*, *Crygb*, *Crygc*, *Crygd*, *Cryge*, *Crygf*, *Dnase2b*, *Bfsp1*, *Gja3*, *Pxdn*, *Sparc*, *Tdrd7*, etc.) and pathways (e.g., structural constituents of eye lens, lens development in camera-type eye, lens fiber cell differentiation, etc.) associated with lens development and cataracts.

## 2. Materials and Methods

### 2.1. Animals

The University of Delaware Institutional Animal Care and Use Committee (IACUC) reviewed and approved the animal protocols described in this study. The Association for Research in Vision and Ophthalmology (ARVO) statement for the use of animals in ophthalmic and vision research was followed for animal experiments. The strategy for generating *Celf1* lens-specific conditional knockout mice is previously described [9]. Briefly, breeding was set up to generate mice (referred to as *Celf1*^cKO^) carrying one *Celf1* germline knockout allele, (referred to as *Celf1^lacZKI^*), one *Celf1* conditional knockout allele (exon five flanked by *loxP* sites, referred to as *Celf1^flox^*) and the lens Cre deleter mouse line P0-3.9GFPCre (The Jackson Laboratory: 024578; henceforth referred to as *Pax6GFPCre*) that initiates Cre expression in the lens placode at embryonic day E9.5 [8,9,29]. GFPCre protein is detected to be highly and predominantly expressed in cells of the lens and pancreatic lineage in this deleter line [29,30], which has been used for generating lens-conditional knockout [8,9,10,29,31]. In the past, mice heterozygous for the *Pax6GFPCre* allele were not found to exhibit any lens defects and were used as a control [9]. *Celf1^flox^* mice without the *Cre* allele were used as a control unless otherwise noted. Briefly, the breeding scheme was as follows. Mice containing *Celf1^lacZKI^* allele were crossed with *Pax6GFPCre* transgenic mouse line to generate *Pax6GFPCre*:*Celf1^lacZKI^*. These were in turn crossed with mice homozygous for the *Celf1* allele in *loxP* sites flank exon 5 (*Celf1^flox/flox^*) to generate mice that carried one allele of Pax6GFPCre, one allele of *Celf1^lacZKI^* and one allele of *Celf1^flox^*. These mice were of mixed backgrounds with contributions from C57BL/6 and FVB strains. Plugs were checked and the day of birth was designated as postnatal day 0 (P0).

### 2.2. Lens RNA Isolation

Lens tissue was micro-dissected from the control and *Celf1*^cKO^ mice, flash-frozen on dry ice and stored at −80 °C until further use. Two P0 lenses were pooled per biological replicate, and three biological replicates each were used for the control and *Celf1*^cKO^ mice for RNA isolation using the RNeasy mini kit (Qiagen, Germantown, TN, USA) for RNA-sequencing. For microarray analysis, total RNA was isolated using RNeasy mini kit (Qiagen) from P6 lenses (1 lens per biological replicate) from *Celf1*^cKO^ and control (*Celf1^lacZKI/+^*) mice. RNA quality was evaluated by Bioanalyzer at the University of Delaware and RNA samples with an RNA quality number (RQN) above 8 were considered for microarrays or library preparation and RNA-sequencing.

### 2.3. RNA-Sequencing and Analysis

Total RNA from the control and *Celf1*^cKO^ P0 mouse lens tissue was used for RNA-sequencing (strand-specific, paired-end 150 bp-libraries) using the Illumina HiSeq 2500 sequencer at the University of Kansas Medical Center Genomics Core. FastQC was used to evaluate the quality of raw paired-end reads. The RNA-sequencing data reported here is submitted to the NCBI Gene Expression Omnibus (GEO) database under series GSE227293. Raw sequences were trimmed to remove the adaptor sequence. Trimmed sequences were aligned on to the mouse genome (GRCm38.p6) with the STAR software (STAR(2.7.8a)) [32], and only uniquely mapped reads were retained for downstream analysis. Reads were associated to genes by featureCount (v2.0.0) [33], and only genes with >0.2 CPM (counts per million) were considered for differential gene expression analysis. The R package edgeR [34] was used to identify differentially expressed genes (DEGs), Fold Change (FC) > 1.5 (|logFC| > 0.58), False Discovery Rate (FDR) < 0.05 and an average expression in log2 CPM > 1.0.

### 2.4. Microarray Analysis

Total RNA from *Celf1*^cKO^ and control (*Celf1^lacZKI/+^*) mouse lenses at stage P6 were isolated as stated above and microarray analysis was performed using the MouseWG-6 v2.0 BeadChip platform (Illumina, San Diego, CA, USA) following previously described protocols [9]. The previously unreported microarray data on stage P6 is submitted to NCBI Gene Expression Omnibus (GEO) database under series GSE225303. Previously generated microarray data on *Celf1*^cKO^ and control (wild-type) mouse lenses at stage P0 (deposited in GEO, GSE101393) was also used for comparative analysis in the present study. For these datasets, gene expression was estimated based on the fluorescence signal intensity of probes associated to specific genes. In cases where multiple probes were associated to the same gene, the expression of the gene was calculated as the logarithmic average of the signals from all probes assigned to the gene. Only the probes that had a fluorescence signal intensity significantly higher than background in at least two samples were retained for downstream differential gene expression analysis. The R package edgeR [34] was used to identify DEGs, with FC > 1.5 (|logFC| > 0.58), FDR <0.05 and an average expression signal > 2 5 (LogSignal > 4.6) cut-offs.

### 2.5. Prioritization of DEGs by Cat-Map, iSyTE, Expression in Fiber vs. Epi, and Pathway Analysis

*Celf1*^cKO^ lens DEGs were examined in the context of Cat-Map, iSyTE, preferential expression in lens fiber cells vs. epithelial cells, and pathway analysis as follows.

#### 2.5.1. Cat-Map: Cataract Associated Genes

*Celf1*^cKO^ lens DEGs known to be associated with cataracts were identified by comparing individual gene names (mouse gene name) for DEGs to the 454 genes (human gene name) listed in the database CatMap (vOct 21) [35]. This identified a subset of genes that were differentially expressed in *Celf1*^cKO^ lenses and whose deficiency or alterations were also associated with human cataracts.

#### 2.5.2. iSyTE: Gene Expression Enrichment in the Lens

To determine the expression enrichment score of the *Celf1*^cKO^ lens DEGs in the normal lens—compared to the whole embryonic body (WB)—in different development stages, we used the database iSyTE [36]. iSyTE contains microarray data from normal mouse lenses at different development stages and the WB reference dataset. The lens-enriched expression scores of the *Celf1*^cKO^ lens DEGs were calculated as the maximum expression of the genes at either E10.5, E12.5, E14.5, E16.5, E17.5, E19.5 or P0, normalized by their expression WB. *Celf1*^cKO^ lens DEGs with an expression enrichment score >1.5 (|logFC| > 0.58) were considered to have enriched expression in normal lens development.

#### 2.5.3. *Celf1*^cKO^ DEGs Preferentially Expressed in Normal Lens Fiber Cells or Epithelial Cells

To identify *Celf1*^cKO^ DEGs preferentially expressed in either normal lens fiber cells (FCs) or the anterior epithelial lens (AEL; also referred to as lens epithelial cells) previously generated data on these lens cell types was used [37]. This data is based on RNA-seq analysis on wild-type (WT) mice at stage P0, which identified 3516 and 3975 genes to be preferentially expressed in FCs and AEL, respectively, based on cut-offs of padj < 0.05 and FC > 1.5.

#### 2.5.4. *Celf1*^cKO^ DEGs Independently Identified as RNA Targets of CELF1 Protein by CLIP-Seq in a Human Cell Line

To identify the subset of *Celf1^cKO^* DEGs directly regulated by CELF1 protein, we examined previously generated crosslinked immunoprecipitation coupled with RNA-sequencing (CLIP-seq) data using CELF1 antibody on the human cell line Hela [38]. In this study, RNAs encoded by 3025 human genes that are bound in cellulo by the CELF1 protein have been identified by CLIP-seq in human Hela cells. We found 2825 (93.4%) mouse orthologs corresponding to these identified targets. Comparative analysis was done between these orthologs and the *Celf1^cKO^* lens DEGs to identify genes that are recognized as RNA targets of CELF1 proteins.

#### 2.5.5. Gene Ontology (GO) Term and Pathways Analysis

The R package ClusterProfiler (v3.18.0) [39] was used to identify Gene Ontology (GO) terms enriched in *Celf1*^cKO^ DEGs by GO enrichment analysis as well as gene set enrichment analysis (GSEA), GO biological process (BP), GO cellular component (CC), and molecular functions (MF). GO analysis in turn led to insights into specific pathways that are altered due to Celf1 deficiency in the lens.

#### 2.5.6. Immunostaining Analysis

Immunostaining was performed as previously described [11,40]. Briefly, mouse embryonic head tissue from stage E16.5 was fixed in 4% paraformaldehyde (PFA) (prepared in 1× phosphate buffer saline, PBS) for 30 min on ice, followed by transfer to 30% sucrose overnight at 4 °C. Once the tissue settled at the bottom, indicating that it was equilibrated, it was mounted in OCT (Tissue Tek, Torrance, CA, USA), frozen and stored at −80 °C until cryosectioning. Cryostat was used to obtain sections of 16 µm thickness. For immunostaining, sections were blocked in a solution of 5% chicken serum, 1% BSA, 0.1% Tween (prepared in 1× PBS) for 1 h at room temperature (RT). The section was subjected to primary antibody (Cryg antibody, Santa Cruz Biotechnology #sc-22415, at 1:100 dil. in 5% chicken serum; E-cad antibody, Cell Signaling #4065, at 1:100 dil. in 5% chicken serum) by overnight incubation at 4 °C. On the following day, slides were washed three times with 1× PBS and incubated for 1 h at RT with the secondary antibody, chicken anti-goat IgG conjugated to Alexa Fluor 488 (1:200 dil.) or anti-rabbit IgG conjugated to Alexa Fluor 594 (1:200 dil.) (Life Technologies, Carlsbad, CA, USA) with the nuclear stain DRAQ5 (1:2000 dil.) (Biostatus Limited, Leicestershire, UK). Slides were washed three times in 1× PBS, mounted and imaged using Zeiss LSM 780 confocal configured with Argon/Krypton laser (488 nm and 561 nm excitation lines) and Helium Neon laser (633 nm excitation line) (Carl Zeiss Inc., White Plains, NY, USA). Adobe Photoshop CS6 (Version: 13.0.0) was used for adjustment of brightness/contrast applied consistently for all images.

## 3. Results

### 3.1. Generation of RNA-Seq Datasets from Celf1^cKO^ and Control Lenses

**Figure 1:**
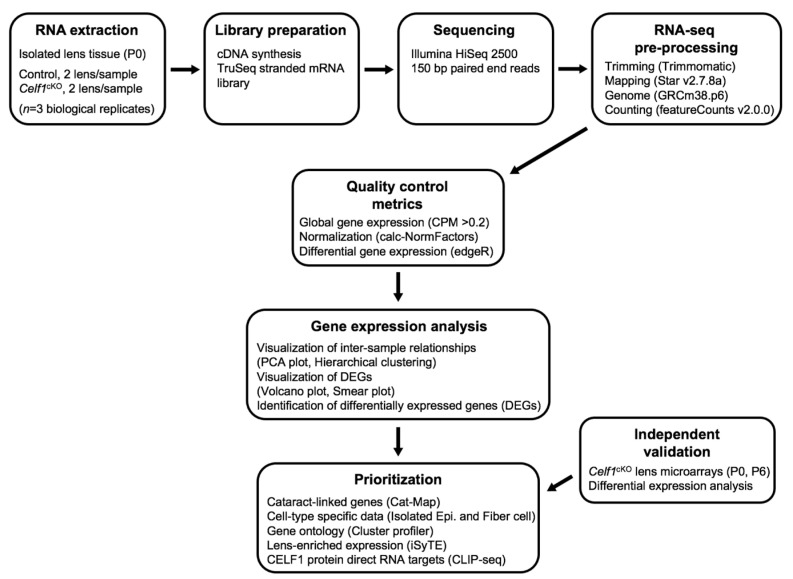
RNA-sequencing (RNA-seq) analysis flowchart. A flowchart outlining the experimental design and bioinformatics pipeline to determine differentially expressed genes between control and *Celf1^cKO^* lenses and their downstream analysis.

Lenses were micro-dissected, and RNA was isolated from stage P0 *Celf1*^cKO^ and control mice as described in detail in the Methods section. An experimental and computational pipeline was developed for RNA-seq analyses (Figure 1). Paired-end, 150 bp-long libraries were prepared, sequenced and analyzed using this strategy. For control and *Celf1*^cKO^ samples, an average of 55.1 million reads per replicate were obtained and aligned using STAR software (STAR(2.7.8a)) [32] (Table S1). On average, 77.3% of the reads were uniquely mapped to the *Mus musculus* reference genome (GRCm38.p6) (Table S1).

### 3.2. Quality Control of RNA-Seq Datasets

**Figure 2:**
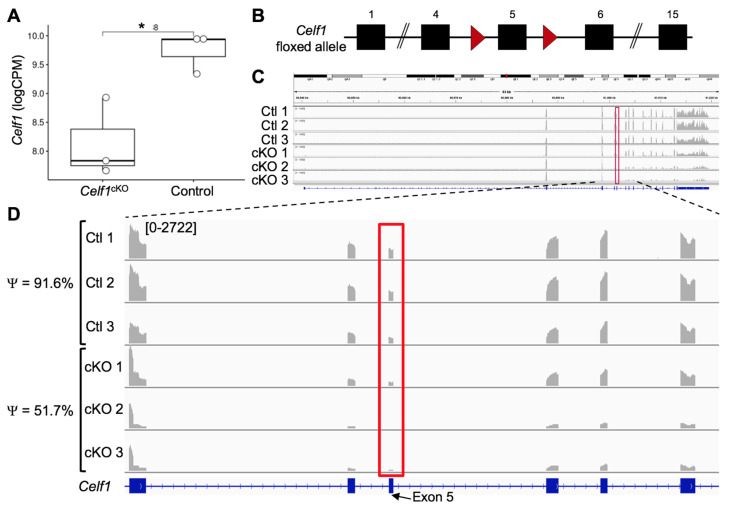
RNA-seq confirms reduction of *Celf1* mRNA in *Celf1^cKO^* mouse lenses. (**A**) Celf1 mRNA levels in logCPM (counts per million) are significantly reduced in *Celf1^cKO^* lenses compared to control (*n* = 3) as estimated by Student’s *t*-test (asterisk indicates *p* < 0.05). (**B**) Schematic of *Celf1* floxed allele showing exon 5 flanked by loxP sites (red arrowheads). (**C**) Visualization of the mapped reads on mouse *Celf1* locus, which at high magnification (**D**) shows that, compared to control lens samples 1–3 that exhibit inclusion of *Celf1* exon 5 in an average of 91.6% of transcripts (represented by Ψ), *Celf1^cKO^* lens samples 1–3 show an average of only 51.7% of transcripts include *Celf1* exon 5 (represented by Ψ). The proportion of transcripts containing exon 5 is estimated by exon junction analysis.

**Figure 3:**
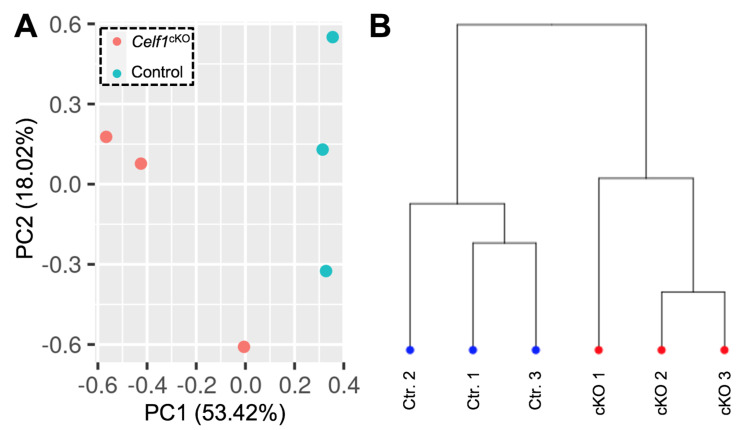
Validation of replicates for control and *Celf1^cKO^* lens RNA-seq datasets. (**A**) Principal component analysis (PCA) of RNA-seq samples shows principal component 1 (PC1) segregates the control replicates from *Celf1^cKO^* replicates. PC1 is responsible for 53.42% of the variance. (**B**) The control and *Celf1^cKO^* replicates can be segregated as per hierarchical clustering analysis based on expression of all genes.

We first examined the Celf1 transcript profiles in *Celf1*^cKO^ and control lenses by visualization of the RNA-seq mapped reads using the software IGV (2.8.10) (mm10) [41] and found Celf1 mRNA to be reduced in *Celf1*^cKO^ lenses (Figure 2A, Table S2). Since the conditional knockout strategy involves removal of exon 5 (Figure 2B), we quantified the inclusion of exon 5 in Celf1 mRNA in *Celf1*^cKO^ and control lenses. It is expected that exon 5 will be deleted by Cre recombinase driven by the *Pax6GFPCre* allele only in *Celf1*^cKO^ lenses, which in turn will result in a premature stop codon. This analysis shows that while control lenses had normal inclusion of exon 5, on average, *Celf1*^cKO^ lenses had 48.3% reduced *Celf1* transcripts that contained exon 5 (Figure 2C,D). Together, these data validate Cre-mediated deletion of *Celf1* in *Celf1*^cKO^ lenses. To assess the quality of the datasets on the global level, principal component analysis (PCA) was performed, which showed that control replicate samples clustered together and separately from *Celf1*^cKO^ replicate samples (Figure 3A). Additionally, hierarchical clustering between samples clearly separated control replicates from *Celf1*^cKO^ replicates (Figure 3B). Together, these analyses validate that Cre-mediated recombination of the *Celf1* conditional knockout allele driven by the *Pax6GFP* Cre-deleter line resulted in global transcriptome changes in the *Celf1*^cKO^ lens. This further confirmed that although Cre-deletion did not result in all *Celf1* transcripts being devoid of exon 5 in *Celf1*^cKO^ lenses, it was sufficient to generate transcriptome changes that result in lens defects.

### 3.3. Identification of Differentially Expressed Genes (DEGs) in Celf1^cKO^ Lens

**Figure 4:**
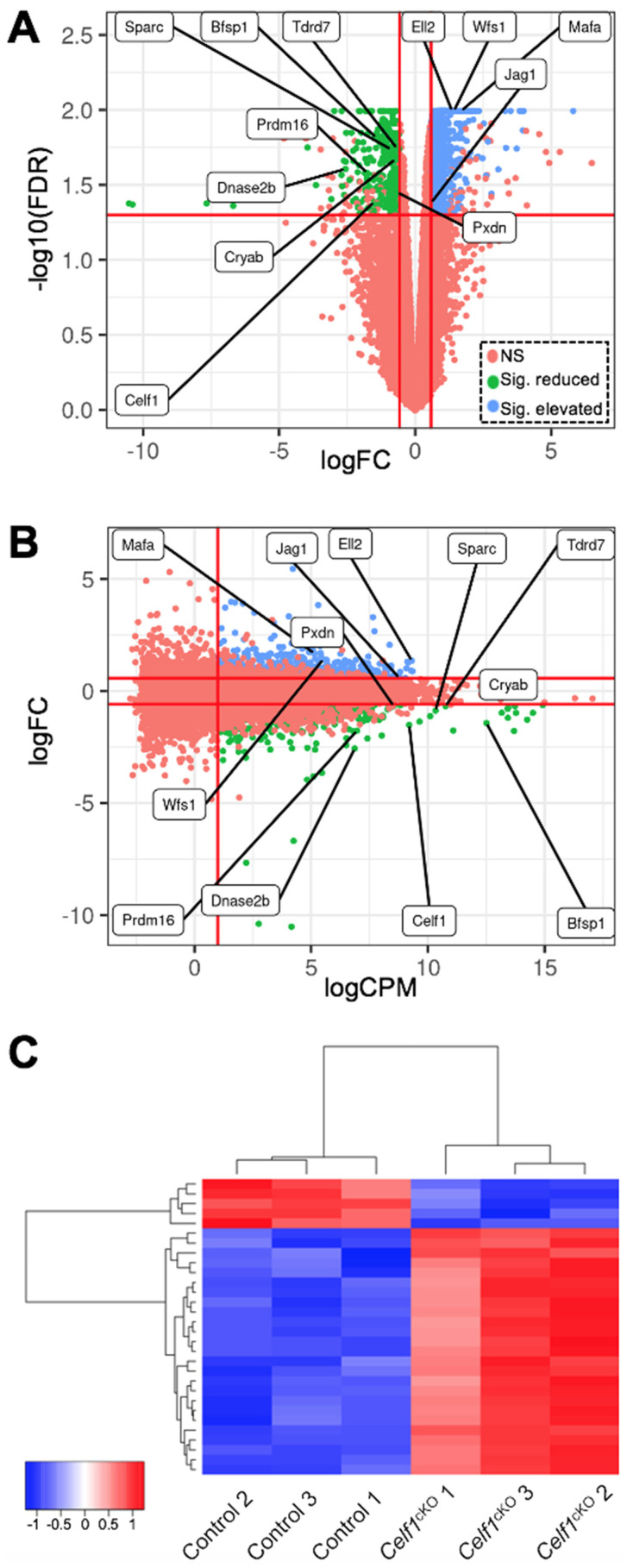
Identification of differentially expressed genes (DEGs) in *Celf1^cKO^* lens. (**A**) Volcano plot and (**B**) smear plot show the different cut-offs used to identify DEGs between *Celf1^cKO^* and control lens RNA-seq samples. With cut-off FDR < 0.05, |logFC| > 0.58, logCPM > 1.0, 660 genes and 327 genes were found to be significantly elevated and reduced, respectively, between *Celf1^cKO^* and control lens samples. NS, not significant. Key cataract/lens-relevant DEGs are labelled in (**A**,**B**). (**C**) Heat map of all significant DEGs in *Celf1^cKO^* lenses compared to control.

Based on cut-off criteria of normalized expression counts >1 log2 counts per million (CPM) averaged across all replicates, >0.58 log2 fold-change and false discovery rate (FDR) < 0.05, a total of 987 differentially expressed genes (DEGs) were identified in *Celf1*^cKO^ lenses, which is visualized by a volcano plot and a smear plot (Figure 4A,B). Of the 987 DEGs, 660 are found to be elevated while 327 are found to be reduced in *Celf1*^cKO^ lenses (Table S2). Further, RNA-seq analysis confirmed the reduction in *Dnase2b* mRNA and elevation of *p21* (*Cdkn1a*) mRNA in *Celf1*^cKO^ lenses (Table S2), as was expected based on our previous findings [9].

### 3.4. Relevance of Celf1^cKO^ Lens DEGs to Lens Development and Cataracts

Next, we sought to prioritize *Celf1*^cKO^ lens DEG candidates that are relevant to lens development and are involved in cataract pathology. Toward this goal, we performed comparative analyses with publicly available datasets relevant to lens biology and pathology. For identifying DEGs linked to cataracts, we used the Cat-Map database [35]. For identifying DEGs exhibiting enriched expression in embryonic lens development, we used the iSyTE database [36]. For identifying DEGs that are preferentially expressed either in the epithelium or fiber cells, we used transcriptome datasets on isolated epithelial and fiber cells [37].

#### 3.4.1. Prioritization of *Celf1*^cKO^ Lens DEGs Using the Cat-Map Database

Comparison of the 987 DEGs with Cat-Map identified 43 genes (including *Celf1*) that are linked to cataracts in humans and/or animal models (Table 1). These genes include several crystallins (e.g., *Cryab*, *Crybb2*, *Cryga*, *etc.*), membrane proteins (e.g., *Gja3*), signaling pathway proteins (e.g., *Jag1*) and other RBPs (e.g., *Tdrd7*). Altered expression of these genes may together contribute to the lens defects observed in *Celf1*^cKO^ mice.

#### 3.4.2. Prioritization of *Celf1*^cKO^ Lens DEGs Using the iSyTE Database

**Figure 5:**
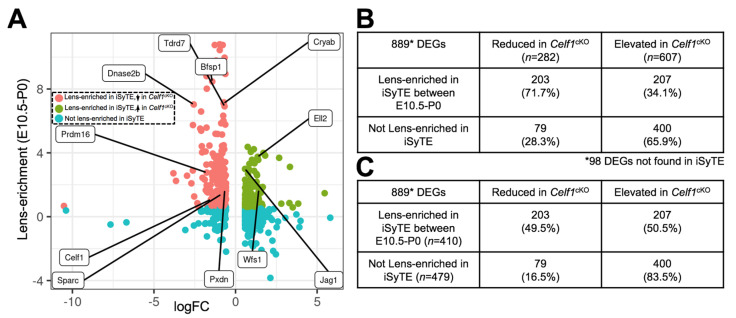
Examination of iSyTE-based lens-enriched expression of *Celf1^cKO^* lens DEGs. (**A**) Quadrant plot of *Celf1^cKO^* lens DEGs (logFC on *x*-axis) and their respective lens-enrichment score between stages E10.5 and P0 as per iSyTE (*y*-axis). The threshold of lens-enrichment is >1.5 fold-change in the lens as per in silico subtraction-based comparison to WB reference dataset. (**B**) Quantification table of reduced and elevated *Celf1^cKO^* lens DEGs with respect to their lens-enrichment score in normal lens development. (**C**) Quantification table, in which percent of lens-enriched DEGs or lens non-enriched DEGs are either reduced or elevated in *Celf1^cKO^* lens DEGs.

The 987 DEGs were examined for their potential lens-enriched expression in iSyTE at stages E10.5, E12.5, E14.5, E16.5, E17.5, E19.5 and P0. Out of 282 reduced genes in the *Celf1*^cKO^ lens, 71.7% (*n* = 203) were found to have lens-enriched expression in at least one of the stages examined (Figure 5A,B; Table S3). In contrast, out of 607 elevated genes in the *Celf1*^cKO^ lens, the majority of the genes 65.9% (*n* = 400) did not exhibit lens-enriched expression (Figure 5A,B; Table S3). Furthermore, when only the DEGs that do not exhibit lens-enriched expression are considered, a vast majority (83.5%) are found to be elevated in iSyTE (Figure 5C). In this analysis, 98 DEGs were not found in iSyTE lens microarray datasets. This may be due to differences between the two transcriptomics approaches (see Discussion). This analysis suggests that Celf1 may contribute to maintaining normal lens developmental transcriptome by negatively regulating genes not normally enriched in the lens as well as positively regulating genes, likely indirectly, which are normally enriched in the lens. This analysis also identified new potential regulators in lens development (e.g., *Ell2*) (Table S3).

#### 3.4.3. Prioritization of *Celf1*^cKO^ Lens DEGs Using Isolated Epithelial and Fiber Cell Transcriptome Data

At early stages of embryonic development, Celf1 exhibits high expression in fiber cells and in later stages is also expressed in epithelial cells [9]. This suggests that it may play a role in transcriptome regulation of both cell types. To examine the impact of Celf1 deficiency in genes preferentially expressed in either epithelial or fiber cells, we compared the 987 DEGs with previously described transcriptome data from isolated epithelial and fiber cells from mouse P0 lenses. First, of the 327 genes reduced in *Celf1*^cKO^ lenses, the majority of the genes (72.5%, *n* = 237) were found to be preferentially expressed in either fiber or epithelial cells (Table 2; Table S4). Of these, the majority of the genes (55.6%, *n* = 182) were preferentially expressed in fiber cells (Table 2; Table S4). In contrast, 16.8% of reduced genes (*n* = 55) were preferentially expressed in epithelial cells. Next, of the 660 genes elevated in *Celf1*^cKO^ lenses, the majority of the genes (52.7%, *n* = 348) did not show preferential expression in either fiber cells or epithelial cells. Of these, the majority of the genes (32.0%, *n* = 211) were preferentially expressed in fiber cells compared to epithelial cells (15.3%, *n* = 101). These data indicate that a deficiency of Celf1 has a substantial impact on transcripts expressed in both fiber cells and epithelial cells. However, the extent of Celf1′s impact is greater on fiber cells compared to epithelial cells. Indeed, independent validation by immunostaining shows that the fiber cell-enriched gamma crystallins are reduced in *Celf1*^cKO^ lenses, in agreement with RNA-seq analysis (Figure S1). Finally, similar to iSyTE data analysis, the upregulated genes in *Celf1*^cKO^ lenses appear to not be enriched in either epithelial or fiber cells in normal lens development. Further, the majority of the elevated genes that are not enriched in epithelial or fiber cells (52.7%, *n* = 348) are also not enriched in iSyTE, suggesting that in normal lens development, Celf1 is necessary, either directly or indirectly, to repress the expression of these genes.

#### 3.4.4. Prioritization of *Celf1*^cKO^ Lens DEGs Using CLIP-Seq Data Identifying Direct RNA Targets of CELF1 Protein in a Human Cell Line

*Celf1* encodes a protein containing three RRMs that enable it to bind to its target RNAs and mediate post-transcriptional regulation of gene expression. Previously, CLIP-seq analysis with a CELF1 antibody has been applied to identify the direct-bound RNA targets of CELF1 protein in the human cell line, Hela [38]. Comparative analysis showed that 32.2% (*n* = 318) of the 987 *Celf1*^cKO^ lens DEGs are also identified in this CLIP-seq dataset. Further, within these 318 DEGs that are directly bound by CELF1 protein, the majority (83.0%; *n =* 264) are found to be significantly elevated in *Celf1*^cKO^ lenses. While 21 of these 318 *Celf1*^cKO^ lens DEGs were not found in iSyTE, 32.4% (*n =* 103) of these DEGs are found to exhibit enriched expressed in normal lenses in iSyTE, while 61.0% (*n* = 194) are not lens-enriched (Table 3). Of the 318 DEGs, the majority are not preferentially expressed in either FCs or AEL and are elevated in *Celf1*^cKO^ lenses (*n* = 149) (Table 4). Among the DEGs preferentially expressed in either cell type, the majority are preferentially expressed in FCs (*n* = 104).

### 3.5. Gene Ontology and Pathway Analysis of Celf1^cKO^ Lens DEGs

**Figure 6:**
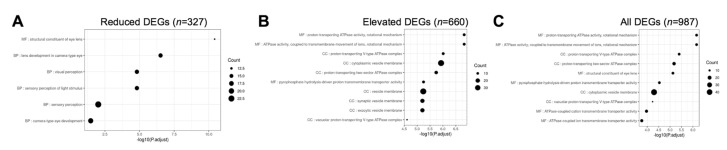
Gene ontology (GO) and pathway analysis on *Celf1^cKO^* lens DEGs obtained from RNA-seq. The top significant GO terms enriched in (**A**) reduced DEGs, (**B**) elevated DEGs, and (**C**) all DEGs in *Celf1^cKO^* lenses.

**Figure 7:**
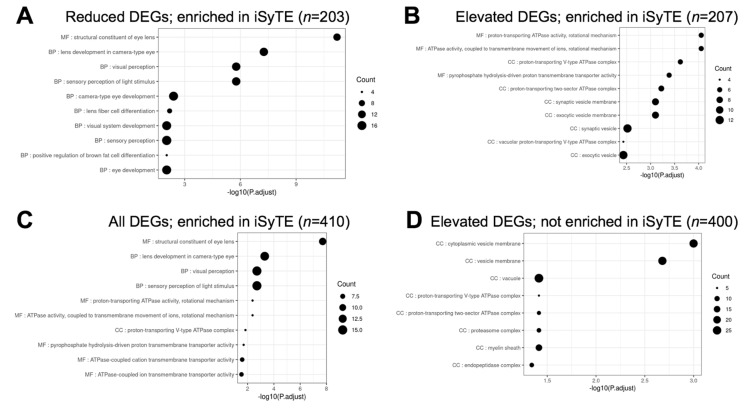
Gene ontology (GO) and pathway analysis on *Celf1^cKO^* DEGs analyzed by iSyTE. The top significant GO terms enriched in (**A**) reduced DEGs, (**B**) elevated DEGs, and (**C**) all DEGs that exhibit enriched expression in normal lenses as per iSyTE. (**D**) The top significant GO terms enriched in *Celf1^cKO^* lens elevated DEGs that do not exhibit enriched expression in normal lenses.

**Figure 8:**
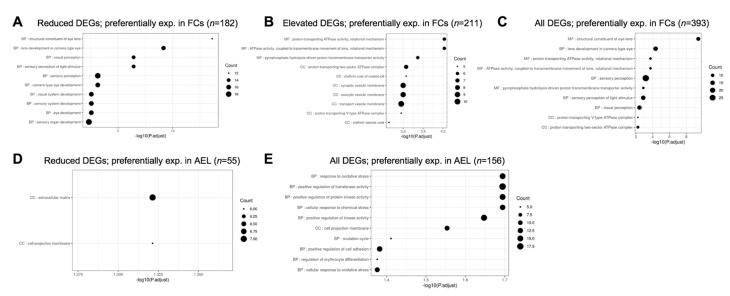
Gene ontology (GO) and pathway analysis on *Celf1^cKO^* lens DEGs preferentially expressed in the anterior epithelium of the lens (AEL) and fiber cells (FCs). The top significant GO terms enriched in (**A**) reduced DEGs, (**B**) elevated DEGs, and (**C**) all DEGs that are preferentially expressed in normal FCs. The top significant GO terms enriched in (**D**) reduced DEGs and (**E**) all DEGs that are preferentially expressed in normal AEL. No significant GO terms were identified in *Celf1^cKO^* elevated DEGs expressed in normal AEL.

**Figure 9:**
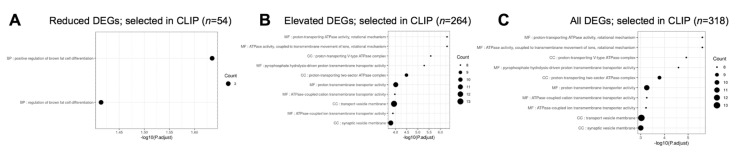
Gene ontology (GO) and pathway analysis on *Celf1^cKO^* lens DEGs that are also identified in CLIP-seq dataset. The top significant GO terms enriched in (**A**) reduced DEGs, (**B**) elevated DEGs, and (**C**) all DEGs that are also identified in CELF1 cross-linked immunoprecipitation followed by RNA-sequencing (CLIP-seq) data on the human cell line (Hela cells).

Next, we examined the different pathways that were represented in *Celf1*^cKO^ lens DEGs. Toward this goal, we performed pathway enrichment analysis by examining gene ontology (GO) enrichment separately on all DEGs, elevated DEGs and reduced DEGs, compared to all the genes expressed in the RNA-seq data. In parallel, GSEA (gene set enrichment analyses) were performed on all the genes in the RNA-seq data that had an expression of at least 1 logCPM and based on their logFC rank. Further, we performed the GI enrichment analysis on a subset of these DEGs that are found to have enriched expression in the lens by iSyTE described in Section 3.4.2. We also performed this analysis on a subset of these DEGs that are preferentially expressed in epithelial or fiber cells described in Section 3.4.3. GSEA analysis was not performed on this subset of DEGs because a large number of genes are required for optimal analysis. This analysis identified “structural constituent of eye lens” (GO:0005212), “lens development in camera-type eye” (GO:0002088), and “visual perception” (GO:0007601) among the top enriched GO terms in 327 reduced DEGs in *Celf1*^cKO^ lenses (Figure 6; Table S5). The same GO terms were identified among reduced DEGs that exhibit enriched expression in normal lens development as per iSyTE (Figure 7) as well as reduced DEGs that are preferentially expressed in normal fiber cells (Figure 8), and these GO terms were also identified by the GSEA analysis as reduced (Table S5). Further, among the reduced DEGs that are preferentially expressed in fiber cells, the GO term “lens fiber cell differentiation” (GO:0070306) was also found to be significantly enriched. These GO categories identified candidate genes with known functions in the lens and/or those associated with cataracts. Among these are several crystallins, *Bfsp1*, *Gja3*, *Tdrd7*, etc. (Table S5). Only two GO terms were found to be enriched among reduced DEGs that are preferentially expressed in epithelial cells. These are “extracellular matrix” (GO:0031012) and “cell projection membrane” (GO:0031253). GSEA analysis and GO enrichment analysis of all the 660 elevated DEGs, or 207 elevated DEGs with enriched expression in normal lens development as per iSyTE, or 211 elevated DEGs preferentially expressed in normal fiber cells, commonly identified, among others, the GO terms, “proton transporting ATPase activity, rotational mechanism” (GO:0046961) and “cytoplasmic vesicle membrane” (GO:0030659) to be enriched. Additionally, in all the 660 elevated DEGs, the GO terms “calcium-dependent protein binding” (GO:0048306), “clathrin coat of coated pit” (GO:0030132), “organelle subcompartment” (GO:0031984) and “protein kinase inhibitor activity” (GO:0004860) were also found to be enriched (Table S5). The majority of the GO terms described above were also identified when all the DEGs or the DEGs with lens-enriched expression, or the DEGs preferentially expressed in fiber cells were considered. Further, of the 453 elevated DEGs that do not have an enriched expression in normal lenses, the GO terms “cytoplasmic vesicle membrane” (GO:0030659), “proton-transporting V-type ATPase complex” (GO:0016471) and “proteasome complex” (GO:0000502) were found to be enriched. Finally, GO term analysis of reduced DEGs that were also identified in CLIP showed enrichment of the terms related to “positive-regulation of brown fat cell differentiation” (GO:0090335) (Figure 9). Among elevated DEGs also identified in CLIP, the GO terms, “translational initiation” (GO:0045948), “clathrin-coated vesicle” (GO:0030136), “ribonucleoprotein complex binding” (GO:0043021) and “calcium-dependent protein binding” (GO:0048306) were identified (Table S5). A subset of these GO terms was also found to be enriched when all DEGs that are identified by CLIP were considered. Thus, GO term analysis identifies pathways whose perturbations contribute to the cataract pathology observed in *Celf1*^cKO^ lenses, which are further highlighted in the Discussion below.

### 3.6. Comparative Analysis of Celf1^cKO^ Lens DEGs Identified by RNA-Seq and Microarrays

**Figure 10:**
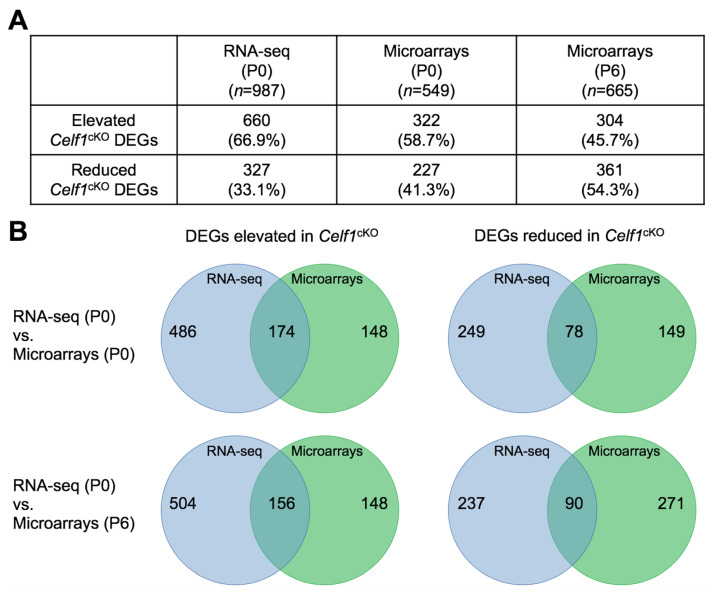
Comparative analysis of *Celf1^cKO^* lens DEGs obtained from RNA-seq and microarrays. (**A**) List of elevated and reduced differentially expressed genes (DEGs) in *Celf1^cKO^* lenses as identified by RNA-seq at stage P0 and microarrays at stage P0 and P6. (**B**) Venn diagrams indicating elevated or reduced *Celf1^cKO^* lens DEGs commonly—or exclusively—identified by RNA-seq and microarray analysis at P0 and P6.

Next, we sought to compare *Celf1*^cKO^ lens DEGs identified by RNA-seq with DEGs that are identified by expression microarrays so as to provide independent validation of the DEGs that can be used for prioritization of candidates. For *Celf1*^cKO^ lenses, published expression microarray data is available for stage P0. There is also unpublished expression microarray data on *Celf1*^cKO^ lenses for stage P6. We first performed differential expression analysis on *Celf1*^cKO^ lens microarray data for stage P0 and P6. This analysis identified 549 DEGs at P0 and 665 DEGs at P6 (Figure 10 and Figure S2; Table S6). Of these, 322 were elevated and 227 were reduced at P0, while 304 were elevated and 361 were reduced at P6. Comparative analysis identified 174 DEGs to be commonly elevated and 78 DEGs to be commonly reduced between RNA-seq and microarrays at P0. Comparative analysis identified 158 DEGs to be commonly elevated and 90 DEGs to be commonly reduced between RNA-seq and microarrays at P6. There is a higher number of DEGs found to be elevated or reduced by the RNA-seq approach at P0. This may be due to technical differences in the two approaches. While microarrays are limited by a predetermined number of genes represented on the array, RNA-seq has no such limitation. Further, while RNA-seq provides individual sequence reads, microarrays depend on probe binding kinetics which may impact their sensitivities. Further, there is a higher number of DEGs found to be mis-expressed by microarrays at P6 compared to P0, which is expected because of the progression of the lens defects. Together, this analysis provides independent validation of numerous DEGs that are mis-expressed upon Celf1 deficiency in the lens (Table S6). Furthermore, among the *Celf1*^cKO^ lens DEGs commonly identified by RNA-seq and microarrays, 84 found at the RNA-seq (P0)–microarray (P0) comparison and 54 found at the RNA-seq (P0)–microarray (P6) comparison were also found to be directly bound by Celf1 protein as per CLIP data (Table S7).

## 4. Discussion

Celf1 encodes an RNA-binding protein that has been associated with various tissue development/cell differentiation and developmental defects/diseases. Celf1 has a role in cells as different as sperm, muscle, and lens cells, among others, and its alterations are associated with various types of cancer and developmental defects including heart defects, myotonic dystrophy and cataracts [6,25,27,42,43,44,45,46,47,48,49]. As an RBP, Celf1 can mediate gene expression control by directly binding to target RNAs and impact their intracellular localization, splicing, stability/decay or translation [38].

Mouse models of *Celf1* deficiency exhibit cataracts and other pathologies [9,10,43]. In the past, different proteins/pathways that are altered due to Celf1-decificiency have been characterized (e.g., p27^Kip1^, Dnase2b, Pax6 and Prox1). For example, previous work described how Celf1 post-transcriptionally controls the dosage of p27^Kip1^ protein by reducing it in fiber cell differentiation, while also being necessary for optimal levels of the nuclease Dnase2b in the lens. Further, Celf1 also functions to negatively control p21^Cip1^ in the lens. Together, these actions of Celf1 result in proper degradation of fiber cell nuclei thereby contributing to optimal refraction of light and lens transparency [9]. Celf1 is also necessary for proper spatiotemporal expression of Prox1 and Pax6 transcription factors in lens development; the disruption of which further contributes to the lens defects [10]. Additionally, an absence of Celf1 is expected to lead to changes to the lens transcriptome that result in lens defects and cataracts. To gain insights into such global perturbations, we performed high-throughput RNA-seq and examined the differentially expressed genes in the *Celf1*^cKO^ lens. While there have been reports on RNA-seq on Celf1 perturbations, in the context of the lens, these are limited to cell lines and not the lens tissue [50,51]. The only transcriptome data available on *Celf1*-deficient lens tissue is on a microarray platform. While microarrays are informative, they have limitations as they depend on probe binding kinetics and can only report on a predefined set of genes. On the other hand, RNA-seq does not present such limitations and offers greater depth of global changes in transcripts.

While the *Celf1*^cKO^ lens exhibits 987 DEGs, interestingly the majority of genes were found to be elevated, suggesting that Celf1 protein—either directly or indirectly—has negative control over these transcripts in normal lenses. However, because 327 were found to be reduced in *Celf1*^cKO^ lenses, this suggests that Celf1 is also necessary for positive control over these genes that may be important for proper lens development. We aimed to identify both, “established” cataract-linked genes as well as potentially novel candidates that are differentially expressed upon Celf1 deficiency. To address the former, we performed analysis of *Celf1*^cKO^ RNA-seq data with respect to the known cataract-linked genes contained in the Cat-Map database. This helped identify the established cataract-linked genes that are significantly impacted because of Celf1 deficiency. On the other hand, the iSyTE database informs on both established cataract-linked genes as well as novel genes that are relevant to lens biology. Therefore, we also performed comparative analysis of *Celf1*^cKO^ RNA-seq data with respect to the genes recognized as lens-enriched in the iSyTE database. Indeed, in addition to identifying known cataract-linked genes, this analysis also identified novel genes with potential functions in the lens. We elaborate below on these findings.

Among the DEGs, 43 genes, including *Celf1*, have previously been linked to cataracts in humans or animal models as per Cat-Map. The majority of these (>60%) were found to be reduced in *Celf1*^cKO^ lenses. This includes *Dnase2b* which is significantly reduced in *Celf1*^cKO^ lenses. Because Dnase2b is necessary for proper nuclear degradation in lens fiber cell differentiation [52] and was also previously found to be a direct RNA target of Celf1 protein in the lens [9], this finding renders confidence in the RNA-seq data. Additionally, several other genes linked to human cataracts were found to be significantly reduced in *Celf1*^cKO^ lenses. These include the crystallins *Cryab*, *Cryba2*, *Cryba4*, *Crybb1*, *Crybb2*, *Cryga*, *Crygb*, *Crygc* and *Crygd*, the connexins *Gjb6* and *Gja3*, the membrane protein *Bfsp1*, the extracellular matrix associated peroxidase *Pxdn*, as well as other post-transcriptional regulatory proteins such as *Tdrd7* [35]. Interestingly, *Celf1*^cKO^ lenses also exhibit significant reduction of *Sparc*, whose deficiency is known to cause cataracts in mice [53]. Because the majority of these key cataract-linked genes are preferentially expressed in fiber cells, this suggests that Celf1 has a major function in controlling fiber cell transcriptome. This finding also suggests that significant reduction of these cohorts of cataract-linked genes may contribute to the cataract pathology in *Celf1*^cKO^ lenses. Furthermore, 16 DEGs that are associated with cataracts were found to be elevated in *Celf1*^cKO^ lenses. This suggests that Celf1 is necessary for optimal transcript levels of genes (neither too high, nor too low) that are critical for lens transparency.

While Cat-Map allows identification of a subset of DEGs that are known to be associated with cataracts, to gain further insights into the impact of *Celf1* deficiency on transcripts relevant to lens biology, comparative analysis was performed using iSyTE. This allowed identification of DEGs that exhibit enriched expression in normal lenses, which has previously been found to be predictive of functions in the lens [30,36,54,55,56]. Thus, mis-regulation of such candidates can potentially contribute to lens pathology. The majority of the genes that are reduced upon *Celf1*-deficiency are found to exhibit enriched expression in normal lens development. Thus, it can be hypothesized that the sub-optimal expression levels of these lens-enriched transcripts could contribute to the cataract pathology. Interestingly, the majority of the DEGs that are elevated in *Celf1*^cKO^ lenses are not found to have enriched expression in normal lens development. Thus, this suggests that elevated expression of these transcripts upon *Celf1*-deficiency may contribute to alterations in lens development.

While iSyTE lens-enrichment is helpful, iSyTE data is primarily based on whole lenses. Celf1 is known to be highly expressed in fiber cells, but later in development it is also known to be expressed in epithelial cells, suggesting that it may have a function in both cell types in the lens [9,10]. Therefore, *Celf1*^cKO^ lens DEGs were examined for their preferential expression in normal isolated lens epithelial or fiber cells. The majority of the DEGs were found to be not preferentially expressed in either cell type and were also found to be elevated in *Celf1*^cKO^ lenses. Thus, similar to the iSyTE lens-enrichment analysis, the cell-type (gene expression in either epithelial or fiber cells) specific analysis reinforces the hypothesis that this subset of non-enriched, elevated *Celf1*^cKO^ DEGs should not be expressed at such high transcript levels for proper lens development. Apart from the DEGs that do not exhibit cell type-preferred expression, the majority of the remaining DEGs are preferentially expressed in fiber cells. This suggests that Celf1′s impact on the lens is primarily through its function in fiber cells. However, it should be noted that *Celf1*^cKO^ DEGs were identified from whole lens samples, which are expected to have higher levels of transcripts from fiber cells compared to epithelial cells. Thus, the sensitivity toward examination of epithelial DEGs is comparatively low. In future, this can be addressed by performing spatial transcriptomics, for example, by conducting RNA-seq on isolated epithelial and fiber cells from *Celf1*^cKO^ lenses. Alternately, this can also be addressed by performing single cell RNA-seq analysis on *Celf1*^cKO^ lenses.

The above analyses inform on the overall impact of Celf1 on normal lens development. To gain insights into the subset of DEGs that are potential direct RNA targets of Celf1 protein, comparative analysis was performed with CLIP-seq data on CELF1 protein in a human cell line. Although this data is from humans, and not mice, and from a non-lens cell line, this analysis identified 32% of *Celf1*^cKO^ lens DEG transcripts to be directly bound by the CELF1 protein in cellulo. The majority of these direct RNA targets of Celf1 protein are found to be elevated in the *Celf1*^cKO^ lens. This supports the hypothesis that Celf1 protein is necessary to directly bind and repress the expression of hundreds of RNAs that are not highly enriched in the normal lens. Among the cell-type preferentially expressed DEGs that are also identified in CLIP-seq, the majority are preferentially expressed in fiber cells, suggesting that the direct impact of Celf1 is higher in the lens fiber cells compared to lens epithelial cells.

Of the *Celf1*^cKO^ lens total DEGs, 32% represent a high number of Celf1-direct RNA targets identified, especially considering that this cell line is not lens-derived and thus may not optimally represent lens gene expression, in addition to other caveats such as the suboptimal expression of Celf1 accessory protein/RNA. Thus, it can be hypothesized that the number of direct DEG RNA targets of the Celf1 protein in the lens may be even higher. This can be addressed in the future by performing CLIP-seq on lens cell lines or whole lens tissue. Further, among the direct RNA targets of Celf1 identified by CLIP-seq, is the transcription elongation factor for RNA polymerase II 2 (Ell2), which exhibits highly enriched expression in normal lens development as per iSyTE. Interestingly, Ell2 expression is significantly elevated in *Celf1*^cKO^ lenses, suggesting that Celf1 protein may function to achieve optimal transcript levels of this key regulatory protein, which is involved in transcription control. Thus, this analysis gives new insights into the specific Celf1 targets that are common regardless of the difference in these cell types (lens vs. Hela) and furthermore are also indicative of the similarities in Celf1 function across different species, namely, mouse and human.

To identify pathways that are altered upon Celf1-deficiency, GSEA analysis and GO term analysis was performed on *Celf1*^cKO^ lens total DEGs, as well as the subset of DEGs prioritized by different approaches. Broadly, DEGs reduced in *Celf1*^cKO^ lenses were found to be enriched in pathways that are relevant to lens development (e.g., “structural constituent of eye lens” (GO:0005212) and “lens development in camera-type eye” (GO:0002088)), while the elevated DEGs represented pathways not enriched in normal lenses (e.g., “proton transporting ATPase activity, rotational mechanism” (GO:0046961)*,* “cytoplasmic vesicle membrane” (GO:0030659) and “calcium-dependent protein binding” (GO:0048306)). Additionally, the GO term “lens fiber cell differentiation” (GO:0070306) was enriched for DEGs that are reduced upon Celf1-deficiency and are also preferentially expressed in normal fiber cells, suggesting that key fiber cell expressed genes are under positive control of Celf1. Interestingly, the GO terms “extracellular matrix” (GO:0031012) and “cell projection membrane” (GO:0031253) were enriched for DEGs preferentially expressed in normal epithelial cells, suggesting that Celf1 may have a distinct role in positive regulation of these processes in epithelial cells. Finally, the GO term “positive-regulation of brown fat cell differentiation” (GO:0090335) was enriched in the subset of reduced DEGs that are direct RNA targets of Celf1 protein. This GO term contained the transcription factor Prdm16, which is independently found to exhibit high lens-enriched expression in iSyTE, especially at/beyond secondary fiber cell differentiation at E16.5. Thus, alteration of *Prdm16* expression in *Celf1*^cKO^ lenses, its identification as a direct RNA target of Celf1 protein, and its enriched expression in normal lenses together make Prdm16 a high-priority candidate whose role in lens development and pathology can be examined in the future.

Together, these various analyses provide insights into lens pathology in *Celf1*^cKO^ mice and identified numerous promising candidates that may be critical for proper lens development. To further prioritize direct RNA targets of Celf1 protein that play a key role in the lens, we used previously reported as well as new microarray transcriptomic analysis on *Celf1*^cKO^ lenses at different postnatal stages (P0 and P6). This allows independent validation of hundreds of DEGs identified by RNA-seq in the *Celf1*^cKO^ lens. Along with the various prioritization approaches described in this report, especially the CLIP-seq analysis that identified direct targets of Celf1 (in addition to other parameters such as, lens-enriched expression in iSyTE, preferential expression in epithelial or fiber cells, etc.), the microarray data identifies high-confidence candidates in the lens for future studies. These analyses show that upon Celf1 deficiency, a cohort of cataract-linked genes are mis-expressed (e.g., the crystallins *Cryab*, *Cryba2*, *Cryba4*, *Crybb1*, *Crybb2*, *Cryga*, *Crygb*, *Crygc* and *Crygd*, the connexins *Gjb6* and *Gja3*, the membrane protein *Bfsp1*, the extracellular matrix associated peroxidase *Pxdn*, and the post-transcriptional regulator *Tdrd7*), in addition to alterations in distinct pathways, thus indicating that multiple factors likely contribute to the manifestation of the cataract defect. Importantly, the present analyses identify as yet unappreciated and novel high-priority candidates in the lens for defining new pathways involved in lens biology (e.g., Ell2 and Prdm16) that likely also contribute to the cataracts resulting from Celf1 deficiency. In particular, the following targets are promising. Ell2, a transcription elongation factor, that functions in a fundamental regulatory process—considered ubiquitously important—in transcription. This is because Ell2 facilitates the release of the RNA Polymerase II from its “pause” in early stages of transcription, which in turn allows the enzyme to proceed with transcription of its target genes. The present study shows that Celf1 functions in controlling the proper dosage of Ell2 in the lens, and thus opens up a new direction in lens research by encouraging the question: are factors like Ell2—that play a critical role in a ubiquitously important regulatory process—specifically recruited for controlling expression of key genes in the lens, a tissue that is known to produce extremely high levels of transcripts that in turn get translated into abundant levels of proteins (e.g., crystallins, which reach concentrations of 450 mg/mL in the lens). Further, this study, by prioritizing Prdm16 which is significantly reduced in Celf1-deficient lenses, has led to the identification of a new transcription factor in the lens, further investigation of which will advance the understanding of gene expression control in this tissue.

## 5. Conclusions

This study reports on the impact of Celf1-deficiency on the early postnatal lens transcriptome. Application of various analyses such as identification in Cat-Map, lens-enriched expression in iSyTE, preferential expression in epithelial or fiber cells, identification as direct RNA target in CLIP-seq data, and GO term enrichment provides insights into key transcriptomic events that are under the control of Celf1 in normal lens development and whose alterations contribute to lens pathology, which includes several established cataract-linked genes such as crystallins, connexins, membrane proteins, etc. Finally, along with independent validation by microarrays, this study provides a new cohort of high-confidence genes (e.g., *Ell2*, *Prdm16*, etc.) for future investigations in lens development.

## Figures and Tables

**Table 1:** *Celf1*^cKO^ lens DEGs linked to cataracts in the Cat-Map database.

Symbol	logFC	logCPM	FDR	Symbol	logFC	logCPM	FDR
*Lgsn*	−3.957	4.826	0.02	*Tdrd7*	−0.673	10.755	0.02
*Gjb6*	−3.08	1.224	0.04	*Cryba4*	−0.671	13.735	0.02
*Dnase2b*	−2.565	6.866	0.03	*Pxdn*	−0.667	8.535	0.04
*Lctl*	−1.985	8.025	0.01	*Flnb*	−0.664	5.836	0.02
*Crybb2*	−1.763	11.087	0.03	*Crybb1*	−0.623	14.92	0.02
*Celf1*	−1.511	9.204	0.04	*Jag1*	0.582	8.78	0.04
*Bfsp1*	−1.428	12.513	0.02	*Psmc3*	0.587	7.955	0.03
*Adgrl2*	−1.326	5.119	0.03	*Ube2a*	0.597	4.673	0.03
*Crygb*	−1.275	14.257	0.02	*Nploc4*	0.6	6.468	0.02
*Cryba2*	−1.195	13.429	0.02	*Sec23a*	0.681	7.201	0.01
*Gja3*	−1.12	10.1	0.02	*Klc1*	0.724	8.255	0.02
*Dnmbp*	−1.109	8.04	0.01	*Ercc6*	0.803	4.453	0.02
*Ulk4*	−1.07	2.037	0.02	*Atm*	0.811	4.526	0.05
*Crygd*	−0.973	14.5	0.01	*Rnf149*	0.857	3.842	0.02
*Cryga*	−0.968	13.166	0.01	*Agps*	0.907	5.422	0.02
*Sparc*	−0.874	10.326	0.02	*Ptn*	1.047	5.652	0.01
*Sord*	−0.815	4.669	0.02	*Polr3b*	1.08	4.807	0.02
*Loxl1*	−0.793	6.509	0.02	*Pqbp1*	1.247	5.673	0.01
*Crygc*	−0.788	13.425	0.01	*Pgrmc1*	1.258	6.345	0.02
*Ace*	−0.785	5.561	0.046	*Wfs1*	1.421	5.521	0.01
*Fkrp*	−0.745	4.324	0.022	*Mafa*	1.684	5.134	0.01
*Cryab*	−0.73	13.439	0.021				

**Table 2:** *Celf1*^cKO^ lens DEGs preferentially expressed in normal lens fiber cells (FCs) or anterior epithelium of the lens (AEL).

Total DEGs (*n* = 987)	Reduced in *Celf1*^cKO^ Lens (*n* = 327)	Elevated in *Celf1*^cKO^ Lens (*n* = 660)
Preferentially exp. in FCs	182	211
Preferentially exp. in AEL	55	101
Not preferentially exp. in FCs or AEL	90	348

**Table 3:** *Celf1*^cKO^ lens DEGs selected by cross-linked immunoprecipitation (CLIP) that exhibit enriched expression in normal lenses as per iSyTE.

DEGs Selected by CLIP (*n* = 297 ^1^)	Reduced in *Celf1*^cKO^ Lens (*n* = 50)	Elevated in *Celf1*^cKO^ Lens (*n* = 247)
Lens enriched-exp. in iSyTE	26	77
Not lens enriched-exp. in iSyTE	24	170

^1^ 21 of the DEGs selected by CLIP were not found in iSyTE.

**Table 4:** *Celf1*^cKO^ lens DEGs selected by cross-linked immunoprecipitation (CLIP) that are preferentially expressed in normal lens fiber cells (FCs) or anterior epithelium of the lens (AEL).

DEGs Selected by CLIP (*n* = 318)	Reduced in *Celf1*^cKO^ Lens (*n* = 54)	Elevated in *Celf1*^cKO^ Lens (*n* = 264)
Preferentially exp. in FCs	30	74
Preferentially exp. in AEL	9	41
Not preferentially exp. in FCs or AEL	15	149

## Data Availability

The RNA-sequencing data and the microarray data described here will be submitted to the Gene Expression Omnibus.

## Supplementary Materials

The following are available online at https://www.mdpi.com/article/10.3390/cells12071070/s1, Figure S1: *Celf1*^cKO^ lens exhibits reduced gamma crystallin expression, Figure S2: Volcano plot and smear plot of *Celf1*^cKO^ lens microarray datasets at P0 and P6, Table S1: Summary of RNA-seq reads and mapping, Table S2: Differential gene expression analysis of *Celf1*^cKO^ lens by RNA-seq, Table S3: iSyTE lens-enriched expression of *Celf1*^cKO^ lens DEGs identified by RNA-seq, Table S4: Epithelial- and fiber cell-preferred expression of *Celf1*^cKO^ lens DEGs identified by RNA-seq, Table S5: GSEA analysis of *Celf1*^cKO^ lens RNA-seq data, Table S6: Gene ontology analysis of *Celf1*^cKO^ lens DEGs, Table S7: Differential gene expression analysis of *Celf1*^cKO^ lens by microarrays at P0 and P6, Table S8: *Celf1*^cKO^ lens DEGs commonly identified by RNA-seq and microarrays.

## References

[1] Harland R.M., Grainger R.M. (2011). Xenopus Research: Metamorphosed by Genetics and Genomics. Trends Genet..

[2] Graw J. (2009). Mouse Models of Cataract. J. Genet..

[3] Shiels A., Hejtmancik J.F. (2021). Inherited Cataracts: Genetic Mechanisms and Pathways New and Old. Exp. Eye Res..

[4] Cvekl A., Zhang X. (2017). Signaling and Gene Regulatory Networks in Mammalian Lens Development. Trends Genet..

[5] Lachke S.A., Maas R.L. (2010). Building the Developmental Oculome: Systems Biology in Vertebrate Eye Development and Disease. Wiley Interdiscip. Rev. Syst. Biol. Med..

[6] Lachke S.A. (2022). RNA-Binding Proteins and Post-Transcriptional Regulation in Lens Biology and Cataract: Mediating Spatiotemporal Expression of Key Factors That Control the Cell Cycle, Transcription, Cytoskeleton and Transparency. Exp. Eye Res..

[7] Dash S., Siddam A.D., Barnum C.E., Janga S.C., Lachke S.A. (2016). RNA-Binding Proteins in Eye Development and Disease: Implication of Conserved RNA Granule Components. Wiley Interdiscip. Rev. RNA.

[8] Dash S., Dang C.A., Beebe D.C., Lachke S.A. (2015). Deficiency of the RNA Binding Protein Caprin2 Causes Lens Defects and Features of Peters Anomaly. Dev. Dyn..

[9] Siddam A.D., Gautier-Courteille C., Perez-Campos L., Anand D., Kakrana A., Dang C.A., Legagneux V., Méreau A., Viet J., Gross J.M. (2018). The RNA-Binding Protein Celf1 Post-Transcriptionally Regulates P27Kip1 and Dnase2b to Control Fiber Cell Nuclear Degradation in Lens Development. PLoS Genet..

[10] Aryal S., Viet J., Weatherbee B.A.T., Siddam A.D., Hernandez F.G., Gautier-Courteille C., Paillard L., Lachke S.A. (2020). The Cataract-Linked RNA-Binding Protein Celf1 Post-Transcriptionally Controls the Spatiotemporal Expression of the Key Homeodomain Transcription Factors Pax6 and Prox1 in Lens Development. Hum. Genet..

[11] Dash S., Brastrom L.K., Patel S.D., Scott C.A., Slusarski D.C., Lachke S.A. (2020). The Master Transcription Factor SOX2, Mutated in Anophthalmia/Microphthalmia, Is Post-Transcriptionally Regulated by the Conserved RNA-Binding Protein RBM24 in Vertebrate Eye Development. Hum. Mol. Genet..

[12] Lachke S.A., Alkuraya F.S., Kneeland S.C., Ohn T., Aboukhalil A., Howell G.R., Saadi I., Cavallesco R., Yue Y., Tsai A.C.-H. (2011). Mutations in the RNA Granule Component TDRD7 Cause Cataract and Glaucoma. Science.

[13] Barnum C.E., Al Saai S., Patel S.D., Cheng C., Anand D., Xu X., Dash S., Siddam A.D., Glazewski L., Paglione E. (2020). The Tudor-Domain Protein TDRD7, Mutated in Congenital Cataract, Controls the Heat Shock Protein HSPB1 (HSP27) and Lens Fiber Cell Morphology. Hum. Mol. Genet..

[14] Lorén C.E., Schrader J.W., Ahlgren U., Gunhaga L. (2009). FGF Signals Induce Caprin2 Expression in the Vertebrate Lens. Differentiation.

[15] Nakazawa K., Shichino Y., Iwasaki S., Shiina N. (2020). Implications of RNG140 (Caprin2)-Mediated Translational Regulation in Eye Lens Differentiation. J. Biol. Chem..

[16] Shao M., Lu T., Zhang C., Zhang Y.-Z., Kong S.-H., Shi D.-L. (2020). Rbm24 Controls Poly(A) Tail Length and Translation Efficiency of Crystallin MRNAs in the Lens via Cytoplasmic Polyadenylation. Proc. Natl. Acad. Sci. USA.

[17] Chen J., Wang Q., Cabrera P.E., Zhong Z., Sun W., Jiao X., Chen Y., Govindarajan G., Naeem M.A., Khan S.N. (2017). Molecular Genetic Analysis of Pakistani Families with Autosomal Recessive Congenital Cataracts by Homozygosity Screening. Investig. Ophthalmol. Vis. Sci..

[18] Fernández-Alcalde C., Nieves-Moreno M., Noval S., Peralta J.M., Montaño V.E.F., Del Pozo Á., Santos-Simarro F., Vallespín E. (2021). Molecular and Genetic Mechanism of Non-Syndromic Congenital Cataracts. Mutation Screening in Spanish Families. Genes.

[19] Kandaswamy D.K., Prakash M.V.S., Graw J., Koller S., Magyar I., Tiwari A., Berger W., Santhiya S.T. (2020). Application of WES Towards Molecular Investigation of Congenital Cataracts: Identification of Novel Alleles and Genes in a Hospital-Based Cohort of South India. Int. J. Mol. Sci..

[20] Tan Y.-Q., Tu C., Meng L., Yuan S., Sjaarda C., Luo A., Du J., Li W., Gong F., Zhong C. (2019). Loss-of-Function Mutations in TDRD7 Lead to a Rare Novel Syndrome Combining Congenital Cataract and Nonobstructive Azoospermia in Humans. Genet. Med..

[21] Tanaka T., Hosokawa M., Vagin V.V., Reuter M., Hayashi E., Mochizuki A.L., Kitamura K., Yamanaka H., Kondoh G., Okawa K. (2011). Tudor Domain Containing 7 (Tdrd7) Is Essential for Dynamic Ribonucleoprotein (RNP) Remodeling of Chromatoid Bodies during Spermatogenesis. Proc. Natl. Acad. Sci. USA.

[22] Zheng C., Wu M., He C.-Y., An X.-J., Sun M., Chen C.-L., Ye J. (2014). RNA Granule Component TDRD7 Gene Polymorphisms in a Han Chinese Population with Age-Related Cataract. J. Int. Med. Res..

[23] Choquet H., Melles R.B., Anand D., Yin J., Cuellar-Partida G., Wang W., Hoffmann T.J., Nair K.S., Hysi P.G., 23andMe Research Team (2021). A Large Multiethnic GWAS Meta-Analysis of Cataract Identifies New Risk Loci and Sex-Specific Effects. Nat. Commun..

[24] Bauermeister D., Claußen M., Pieler T. (2015). A Novel Role for Celf1 in Vegetal RNA Localization during Xenopus Oogenesis. Dev. Biol..

[25] Vlasova-St Louis I., Dickson A.M., Bohjanen P.R., Wilusz C.J. (2013). CELFish Ways to Modulate MRNA Decay. Biochim. Biophys. Acta.

[26] Zheng Y., Miskimins W.K. (2011). CUG-Binding Protein Represses Translation of P27Kip1 MRNA through Its Internal Ribosomal Entry Site. RNA Biol..

[27] Ladd A.N., Charlet N., Cooper T.A. (2001). The CELF Family of RNA Binding Proteins Is Implicated in Cell-Specific and Developmentally Regulated Alternative Splicing. Mol. Cell. Biol..

[28] Vlasova I.A., Tahoe N.M., Fan D., Larsson O., Rattenbacher B., Sternjohn J.R., Vasdewani J., Karypis G., Reilly C.S., Bitterman P.B. (2008). Conserved GU-Rich Elements Mediate MRNA Decay by Binding to CUG-Binding Protein 1. Mol. Cell..

[29] Rowan S., Conley K.W., Le T.T., Donner A.L., Maas R.L., Brown N.L. (2008). Notch Signaling Regulates Growth and Differentiation in the Mammalian Lens. Dev. Biol..

[30] Lachke S.A., Ho J.W.K., Kryukov G.V., O’Connell D.J., Aboukhalil A., Bulyk M.L., Park P.J., Maas R.L. (2012). ISyTE: Integrated Systems Tool for Eye Gene Discovery. Investig. Ophthalmol. Vis. Sci..

[31] Rowan S., Siggers T., Lachke S.A., Yue Y., Bulyk M.L., Maas R.L. (2010). Precise Temporal Control of the Eye Regulatory Gene Pax6 via Enhancer-Binding Site Affinity. Genes. Dev..

[32] Dobin A., Davis C.A., Schlesinger F., Drenkow J., Zaleski C., Jha S., Batut P., Chaisson M., Gingeras T.R. (2013). STAR: Ultrafast Universal RNA-Seq Aligner. Bioinformatics.

[33] Liao Y., Smyth G.K., Shi W. (2014). FeatureCounts: An Efficient General Purpose Program for Assigning Sequence Reads to Genomic Features. Bioinformatics.

[34] Chen Y., Lun A.T.L., Smyth G.K. (2016). From Reads to Genes to Pathways: Differential Expression Analysis of RNA-Seq Experiments Using Rsubread and the EdgeR Quasi-Likelihood Pipeline. F1000Research.

[35] Shiels A., Bennett T.M., Hejtmancik J.F. (2010). Cat-Map: Putting Cataract on the Map. Mol. Vis..

[36] Kakrana A., Yang A., Anand D., Djordjevic D., Ramachandruni D., Singh A., Huang H., Ho J.W.K., Lachke S.A. (2018). ISyTE 2.0: A Database for Expression-Based Gene Discovery in the Eye. Nucleic Acids Res..

[37] Zhao Y., Zheng D., Cvekl A. (2018). A Comprehensive Spatial-Temporal Transcriptomic Analysis of Differentiating Nascent Mouse Lens Epithelial and Fiber Cells. Exp. Eye Res..

[38] Le Tonquèze O., Gschloessl B., Legagneux V., Paillard L., Audic Y. (2016). Identification of CELF1 RNA Targets by CLIP-Seq in Human HeLa Cells. Genom. Data.

[39] Yu G., Wang L.-G., Han Y., He Q.-Y. (2012). ClusterProfiler: An R Package for Comparing Biological Themes among Gene Clusters. OMICS.

[40] Agrawal S.A., Anand D., Siddam A.D., Kakrana A., Dash S., Scheiblin D.A., Dang C.A., Terrell A.M., Waters S.M., Singh A. (2015). Compound Mouse Mutants of BZIP Transcription Factors Mafg and Mafk Reveal a Regulatory Network of Non-Crystallin Genes Associated with Cataract. Hum. Genet..

[41] Thorvaldsdóttir H., Robinson J.T., Mesirov J.P. (2013). Integrative Genomics Viewer (IGV): High-Performance Genomics Data Visualization and Exploration. Brief. Bioinform..

[42] Beisang D., Bohjanen P.R., Vlasova-St. Louis I.A., Abdelmohsen K. (2012). CELF1, a Multifunctional Regulator of Posttranscriptional Networks. Binding Protein.

[43] Kress C., Gautier-Courteille C., Osborne H.B., Babinet C., Paillard L. (2007). Inactivation of CUG-BP1/CELF1 Causes Growth, Viability, and Spermatogenesis Defects in Mice. Mol. Cell. Biol..

[44] Cifdaloz M., Osterloh L., Graña O., Riveiro-Falkenbach E., Ximénez-Embún P., Muñoz J., Tejedo C., Calvo T.G., Karras P., Olmeda D. (2017). Systems Analysis Identifies Melanoma-Enriched pro-Oncogenic Networks Controlled by the RNA Binding Protein CELF1. Nat. Commun..

[45] Chaudhury A., Cheema S., Fachini J.M., Kongchan N., Lu G., Simon L.M., Wang T., Mao S., Rosen D.G., Ittmann M.M. (2016). CELF1 Is a Central Node in Post-Transcriptional Regulatory Programmes Underlying EMT. Nat. Commun..

[46] House R.P., Talwar S., Hazard E.S., Hill E.G., Palanisamy V. (2015). RNA-Binding Protein CELF1 Promotes Tumor Growth and Alters Gene Expression in Oral Squamous Cell Carcinoma. Oncotarget.

[47] Matsui T., Sasaki A., Akazawa N., Otani H., Bessho Y. (2012). Celf1 Regulation of Dmrt2a Is Required for Somite Symmetry and Left-Right Patterning during Zebrafish Development. Development.

[48] Timchenko N.A., Cai Z.J., Welm A.L., Reddy S., Ashizawa T., Timchenko L.T. (2001). RNA CUG Repeats Sequester CUGBP1 and Alter Protein Levels and Activity of CUGBP1. J. Biol. Chem..

[49] Philips A.V., Timchenko L.T., Cooper T.A. (1998). Disruption of Splicing Regulated by a CUG-Binding Protein in Myotonic Dystrophy. Science.

[50] Xiao J., Jin S., Wang X., Huang J., Zou H. (2022). CELF1 Selectively Regulates Alternative Splicing of DNA Repair Genes Associated With Cataract in Human Lens Cell Line. Biochem. Genet..

[51] Xiao J., Tian X., Jin S., He Y., Song M., Zou H. (2022). CELF1 Promotes Matrix Metalloproteinases Gene Expression at Transcriptional Level in Lens Epithelial Cells. BMC Ophthalmol..

[52] Nishimoto S., Kawane K., Watanabe-Fukunaga R., Fukuyama H., Ohsawa Y., Uchiyama Y., Hashida N., Ohguro N., Tano Y., Morimoto T. (2003). Nuclear Cataract Caused by a Lack of DNA Degradation in the Mouse Eye Lens. Nature.

[53] Gilmour D.T., Lyon G.J., Carlton M.B., Sanes J.R., Cunningham J.M., Anderson J.R., Hogan B.L., Evans M.J., Colledge W.H. (1998). Mice Deficient for the Secreted Glycoprotein SPARC/Osteonectin/BM40 Develop Normally but Show Severe Age-Onset Cataract Formation and Disruption of the Lens. EMBO J..

[54] Anand D., Lachke S.A. (2017). Systems Biology of Lens Development: A Paradigm for Disease Gene Discovery in the Eye. Exp. Eye Res..

[55] Anand D., Kakrana A., Siddam A.D., Huang H., Saadi I., Lachke S.A. (2018). RNA Sequencing-Based Transcriptomic Profiles of Embryonic Lens Development for Cataract Gene Discovery. Hum. Genet..

[56] Aryal S., Anand D., Hernandez F.G., Weatherbee B.A.T., Huang H., Reddy A.P., Wilmarth P.A., David L.L., Lachke S.A. (2020). MS/MS in Silico Subtraction-Based Proteomic Profiling as an Approach to Facilitate Disease Gene Discovery: Application to Lens Development and Cataract. Hum. Genet..

