# Supplementary figures and images for: High-Throughput Transcriptomics of Celf1 Conditional Knockout Lens Identifies Downstream Networks Linked to Cataract Pathology
Source: Cells. 2023 Apr 1;12(7):1070. doi: 10.3390/cells12071070 (PMC10093462; doi:10.3390/cells12071070)

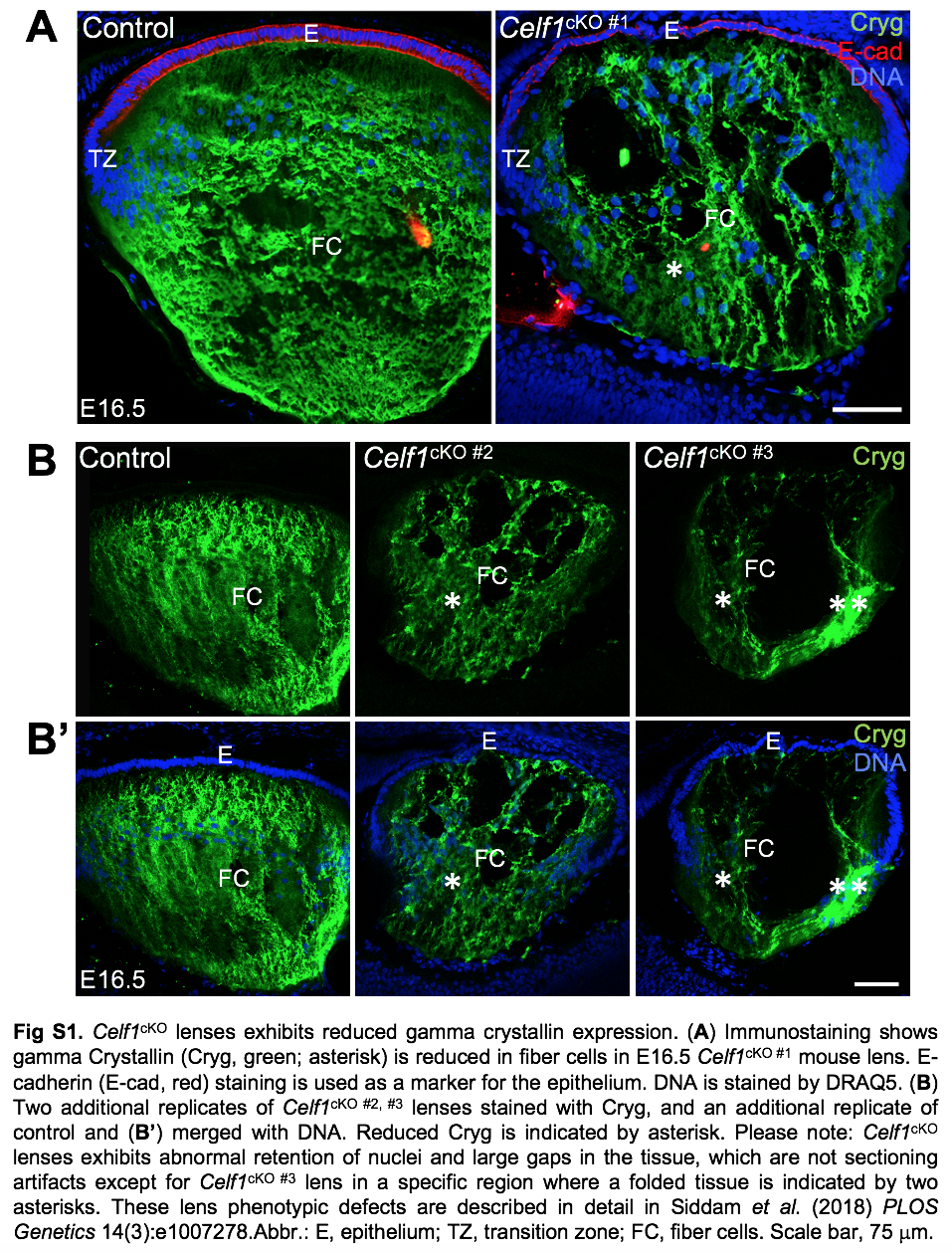

Supplement: Supplementary file 1 [file cells-12-01070-s001.zip › cells-2223860 Supplementary Files Revised Submited March 29 2023/Siddam Fig S1 Revised March 29 2023.tiff]

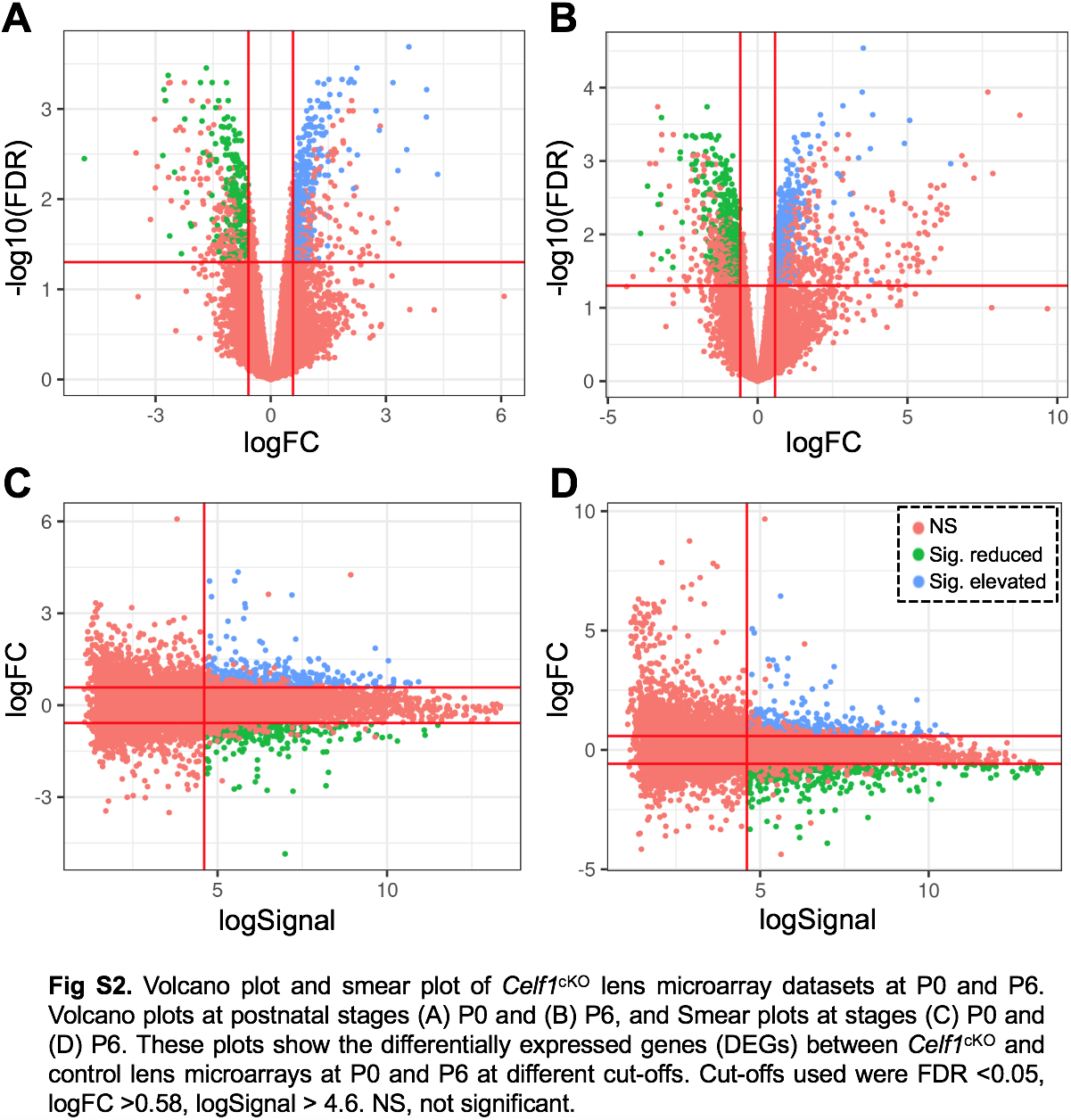

Supplement: Supplementary file 1 [file cells-12-01070-s001.zip › cells-2223860 Supplementary Files Revised Submited March 29 2023/Siddam Fig S2 2023 March 13 Revised.tiff]
